# Health Officers Annual Report of the City of Rochester

**Published:** 1871-08

**Authors:** 


*Health Officers Annual Report of the City of Rochester.* By HARVEY F. MONTGOMERY, M. D.

This is a very excellent report of the Health Physician. We find the following upon summer complaints, which, as it may be interesting to our readers, we make the quotations:—

"Milk is the main article of food for children at the age when they are "cutting their teeth." During the hot months our mortality is greatly increased and this increase is mainly among infants between one and five years of age; these deaths are the result of diarrhea, and imperfect digestion or nutrition. Children in the country, where the milk after being taken from the cow, is immediately carried to, and kept in a cool place till used, do not suffer from "summer complaints;" hence we may infer with Mr. Owen, Milk Inspector of Cincinnati, that putrid milk is an active agent in producing these diseases. I will quote from Inspector Owen's report:—

"The importance of removing animal heat from milk, before sending it to market, cannot well be over estimated in our city. Thousands of children die during the summer months of diseases of the digestive organs, by unwholesome milk alone. There is another condition of milk far more unwholesome than sour milk. It is called tainted milk, and is produced by transporting warm milk during the extremely hot weather to market, in closely covered cans. On opening the cans, a sickly, offensive odor, we might as well call the odor of putrid meat, animal odor arises. The milk is not sour; it has a slimy alkaline taste. Putrefaction has commenced in it. It is putrid and poisonous. It is a startling fact, not generally known, but nevertheless true, that milk brought to this city for sale during the extreme hot weather, confined in close cans, and exposed to the noon-day sun, reeking hot, with the animal heat not removed, is sometimes sold in this putrid state, and is about as wholesome for food, as putrid meat, or rotten eggs. Yet it is used under the mistaken notion that, because it is not sour, it is not unwholesome. Little, if any, of the milk brought to this city is properly cooled; most of it is not cooled at all, and that which is peddled around usually, is not fit to be poured down the throats of helpless infants. No wonder so many die. The wonder is, that so many live. The milk ought to be cooled immediately,—thoroughly cooled."

We find, also, the following suggestion upon the Disinfection of Night Soil:—

"One of the great evils and annoyances of the city, is the "night-cart," which fills the street where it passes, with offensive and unwholesome effluvia. This may be obviated to a certain extent, and I would suggest to the Board of Health to require the scavengers to procure the proper material, and to use it before removing the "night-soil" from the vaults. About two lbs. (or one quart) of liquid sesqui-chloride of iron, full strength, combined with 10 per cent. of its own weight of crude carbolic acid, will disinfect a cubic yard of "night-soil," according to experiments made by order of the Metropolitan Board of Health of New York City.

"This disinfectant is mixed before use, with ten times its volume of water, and then about one-half of this solution is poured upon the contents of the privy, and thoroughly stirred with an implement. About half an hour after this has been done, they begin to empty the privy, gradually using more of the disinfecting material, as they take the "soil" out. The residue is then sprinkled over the walls of the vault to disinfect them thoroughly." This fluid prepared for use, can probably be furnished to the scavengers at a cost of 30 cents for each load to be removed."

